# Sequencing and characterizing the complete chloroplast genome of *Ardisia silvestris* Pit., a potential medicinal plant in Asia

**DOI:** 10.3389/fpls.2025.1627578

**Published:** 2025-09-12

**Authors:** Nhat Nam Nguyen, Hoang Dang Khoa Do

**Affiliations:** ^1^ School of Agriculture and Aquaculture, Tra Vinh University, Vinh Long, Vietnam; ^2^ Functional Genomics Research Center, NTT Hi-Tech Institute, Nguyen Tat Thanh University, Ho Chi Minh City, Vietnam; ^3^ Center for Hi-Tech Development, Saigon Hi-Tech Park, Nguyen Tat Thanh University, Ho Chi Minh City, Vietnam

**Keywords:** comparative genomics, myrsinoideae, plastid genome, plastome evolution, primulaceae

## 1 Introduction

*Ardisia* Sw. 1788 is one of 55 genera of Primulaceae and contains 739 accepted species that distribute in subtropical and tropical areas (Plants of the World Online, 2025). *Ardisia* species contains different phytochemical constituents such as coumarins, ardisiaquinones, and alkylphenols and was used as traditional medicine for fever, inflammation, and cancer (Kobayashi and de Mejía, 2005; de Mejía and Ramírez-Mares, 2011; Liu et al., 2022; Tian-Liang et al., 2024). Specifically, a benzoquinonoid compound was extracted from *Ardisia crispa* and exhibited antimetastatic and antitumor features (Kang et al., 2001). The combination of *Ardisia gigantifolia* leaf extract and silver nanoparticles indicated an anti-cancer activity (Le et al., 2023). *Ardisia silvestris* is native to Vietnam and Hainan (China) and its ethanol extract possessed the characteristics of antiphotoaging and skin-protective activities (Huang et al., 2023; Plant of the World Online, 2025). Additionally, a previous study revealed a notable anti-inflammatory characteristic of *A. silvestris* ethyl acetate extract (Thanh et al., 2025). Also, the antioxidant and antibacterial properties of *A. silvestris* leaf extract (Huynh, 2020). These previous results demonstrated the medicinal values of *A. silvestris* and related species in *Ardisia* genus. However, genomic data, including nuclear, mitochondrial, and chloroplast genomes, of *A. silvestris* are limited and need further investigations.

Chloroplast genome is an essential component in autotrophic plants because it encodes genes responsible for performing photosynthesis (Dobrogojski et al., 2020). The chloroplast genome had a quadripartite structure including a large single copy, a small single copy, and two inverted repeat regions, which could be altered in both autotrophic and heterotrophic plants (Daniell et al., 2016). Additionally, the genomic information of chloroplast genomes reflected the evolutionary history, which was used to explore a billion years of plant evolution (Gitzendanner et al., 2018). Previously, chloroplast genomes of Primulaceae species have been reported (Xu et al., 2020; Xie et al., 2023; Li et al., 2024). The complete chloroplast genomes of various *Ardisia* species such as *A. crispa, A. gigantifolia, A. crenata, A. villosa, A. mamillata, A. brunnescents, A. pusilla, A. squamulosa, A. brevicaulis*, and *A. crenata* were also published (Xie et al., 2021; Ye et al., 2024; Yuan et al., 2024). In the current study, we report the complete chloroplast genome of *Ardisia silvestris*, collected it Vietnam, using the Illumina sequencing flatform. The result of our study enriches the chloroplast genome data of *Ardisia* genus and provides initial chloroplast genomic data for further genomic studies examining phylogeny and molecular markers of *A. silvestris* and related taxa in Primulaceae.

## 2 Materials and methods

### 2.1 Plant sampling, DNA extraction, and next-generation sequencing

The healthy leaves of *Ardisia silvestris* were collected from living collection of medicinal plants at Tra Vinh University, Vinh Long Province, Vietnam (9°55’25.0”N 106°20’52.4”E). Then, the leaves were stored at −80°C in a deep freezer for further experiments. The total genomic DNA was extracted from the frozen leaves of *A. silvestris* using DNeasy Plant Pro Kit (Qiagen, USA) following the manufacturer’s instructions. The quality of DNA sample was checked using NanoDrop One Microvolume UV-Vis Spectrophotometer (Thermo Fisher Scientific, USA) and 1% agarose gel electrophoresis. The DNA sample selected for Nextseq550 sequencing (Illumina, USA) should have a concentration of 100 ng/µL and show a clear band on the agarose gel. The TruSeq DNA Nano kit (Illumina, USA) was used to prepare sequencing library to generate paired-end reads of 150 bp following the manufacturer’s instructions.

### 2.2 Assembly and annotation of chloroplast genome

The raw reads were qualified and filtered using fastp v0.24.1 to remove the adapter sequences and eliminate the reads possessing a Qscore under 20, having length shorter than 100 bp, and containing more than five N bases (Chen et al., 2018). The remaining high-quality reads were then assembled to complete chloroplast genome using NOVOPlasty v4.3.5 with the reference sequence of *Ardisia fordii* (NCBI accession number NC_060707) and other default settings (Dierckxsens et al., 2016). Consequently, the newly completed chloroplast genome of *A. silvestris* was annotated using Geseq through online interface at https://chlorobox.mpimp-golm.mpg.de/geseq.html with default settings (Tillich et al., 2017). To verify the annotation of Geseq, the annotation of protein-coding region was rechecked the start and stop codon of each gene using Geneious Prime v2024.0.1 (https://www.geneious.com/) whereas the structural formation of tRNA regions were tested using tRNAscan-SE 2.0 available at https://lowelab.ucsc.edu/tRNAscan-SE/index.html with default settings (Chan and Lowe, 2019). Additionally, the quadripartite structure of chloroplast genome, including a large single copy, a small single copy, and two inverted repeat regions, was investigated using the “Find repeat” function with the setting of minimum repeat length of 10,000 bp of Geneious Prime v0.2024.1 to locate two inverted repeat regions that flanked the large single copy and the small single copy regions. The map of chloroplast genome was illustrated using OGDRAW v1.3.1 available at https://chlorobox.mpimp-golm.mpg.de/OGDraw.html with default settings for plastid sequences (Greiner et al., 2019). The complete chloroplast genome of *A. silvestris* was deposited to GenBank under accession number PV608499.

## 3 Results

**Figure 1:**
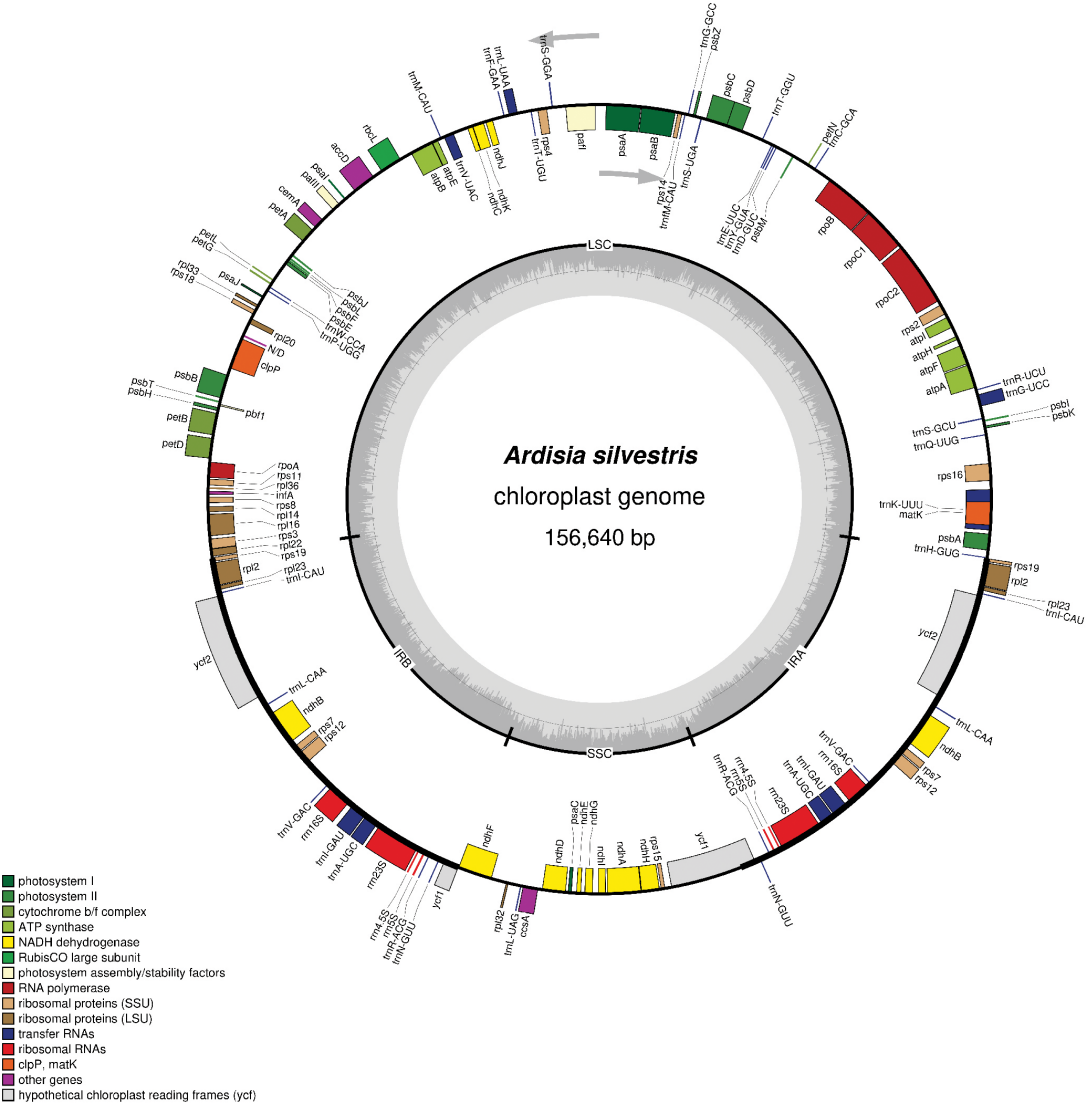
The chloroplast genome map of *Ardisia silvestris*. The arrows indicated the translation directions of inner and outer genes. The inner circle with grey color illustrates GC content. The inner circle indicates four regions of the chloroplast genome. LSC, large single copy; SSC, small single copy; IRA and IRB, inverted repeat regions.

The assembly process resulted in a quadripartite chloroplast genome of *A. silvestris* with a mean coverage of 1642x (**Figure 1**). This genome was 156,640 bp in length and had 37.3% GC content. Additionally, the complete chloroplast genome of *A. silvestris* consisted of a large single copy (LSC) region of 85, 812 bp (35.2% GC content), a small single copy (SSC) region of 18,388 bp (30.4% GC content), and two inverted repeat (IR) regions of 26,220 bp (43.2% GC content) each. Further observation revealed that the junction between LSC and IR regions located within *rps19* coding region whereas that of SSC and IR regions was in the coding region of *ycf1*. The complete chloroplast genome of *A. silvestris* encoded 79 unique protein-coding genes, 30 unique transfer RNA genes, and four unique ribosomal RNA genes (**Table 1**). Among 113 unique coding genes, 19 regions were duplicated in IR region including *rps19, rpl2*, *rpl23*, *trnI_CAU*, *ycf2*, *trnL_CAA*, *ndhB*, *rps7*, *rps12*, *trnV_GAC*, *rrn16*, *trnI_GAU*, *trnA_UGC*, *rrn23*, *rrn4.5*, *rrn5*, *trnR_ACG*, *trnN_GUU*, and *ycf1*. Notably, *ycf1* and *rps19* exhibited incomplete duplication due to expansion of IR regions. Additionally, there were nine protein genes (including *rps16*, *atpF*, *rpoC1*, *petB*, *petD*, *rpl16*, *rpl2*, *ndhB*, and *ndhA*) and six tRNAs (including *trnK_UUU, trnI_GAU*, *trnA_UGC*, *trnG_UCC*, *trnL_UAA*, and *trnV_UAC*) contained one intron. Meanwhile, *pafI* and *clpP1* had two introns. The *rps12* gene was trans-spliced of which the exon 2 and exon 3 located in IR regions.

**Table 1:** Gene composition of *Ardisia silvestris* chloroplast genome.

Groups of genes	Name of genes	Quantity
Ribosomal RNAs	*rrn4.5^a^, rrn5^a^, rrn16^a^, rrn23^a^*	8
Transfer RNAs	*trnA_UGC^a,b^, trnC_GCA, trnD_GUC, trnE_UUC, trnF_GAA, trnG_UCC^b^, trnG_GCC, trnH_GUG, trnI_GAU^a,b^, trnK_UUU^b^, trnL_CAA^a^, trnL_UAA^b^, trnL_UAG, trnfM_CAU, trnI_CAU^a^, trnM_CAU, trnN_GUU^a^, trnP_UGG, trnQ_UUG, trnR_ACG^a^, trnR_UCU, trnS_GCU, trnS_GGA, trnS_UGA, trnT_GGU, trnT_UGU, trnV_GAC^a^, trnV_UAC^b^, trnW_CCA, trnY_GUA*	37
Large units of ribosome	*rpl2^a,b^, rpl14, rpl16^b^, rpl20, rpl22, rpl23 ^a^, rpl32, rpl33, rpl36*	11
Small units of ribosome	*rps2, rps3, rps4, rps7^a^, rps8, rps11, rps12^a^, rps14, rps15, rps16 ^b^, rps18, rps19^a^*	15
RNA polymerase	*rpoA, rpoB, rpoC1^b^, rpoC2*	4
Translational initiation factor	*infA*	1
Subunit of photosystem I	*psaA, psaB, psaC, psaI, psaJ, pafI^c^, pafII*	7
Subunit of photosystem II	*psbA, psbB, psbC, psbD, psbE, psbF, psbH, psbI, psbJ, psbK, psbL, pbfI, psbM, psbT, psbZ*	15
Subunit of cytochrome	*petA, petB^b^, petD^b^, petG, petL, petN*	6
Subunit of ATP synthases	*atpA, atpB, atpE, atpF^b^, atpH, atpI*	6
Large unit of Rubisco	*rbcL*	1
Subunit of NADH dehydrogenase	*ndhA^b^, ndhB^a,b^, ndhC, ndhD, ndhE, ndhF, ndhG, ndhH, ndhI, ndhJ, ndhK*	12
Maturase	*matK*	1
Envelope membrane protein	*cemA*	1
Subunit of acetyl-CoA	*accD*	1
C-type cytochrome synthesis gene	*ccsA*	1
ATP-dependent protease subunit P	*clpP1 ^c^*	1
Hypothetical proteins and conserved reading frames	*ycf1 ^a^, ycf2^a^*	4

^a^duplicated gene in IR region; ^b^genes containing single intron, ^c^genes containing two introns.

## Data Availability

The datasets presented in this study can be found in online repositories. The names of the repository/repositories and accession number(s) can be found below: https://www.ncbi.nlm.nih.gov/genbank/, PV608499 https://www.ncbi.nlm.nih.gov/, PRJNA1261444.
